# Mortality Outcomes of Combined Heart and Liver Transplantation and Isolated Heart Transplantation Following Fontan Procedures: A Systematic Review and Meta‐Analysis

**DOI:** 10.1111/petr.70174

**Published:** 2025-09-07

**Authors:** Oscar Holmvard, Mariana Póvoa‐Corrêa, Adriana Macintyre Innocenzi, Lucio Filgueiras Pacheco, Daniella Braz Parente, Ronir Raggio Luiz, Jessica Pronestino de Lima Moreira, Renata de Mello Perez, Fernanda Padrão Fernandes, Renata Moll‐Bernardes

**Affiliations:** ^1^ D'Or Institute for Research and Education (IDOR) Rio de Janeiro RJ Brazil; ^2^ Maternal and Child Health Department Federal University of Rio de Janeiro (UFRJ) Macaé RJ Brazil; ^3^ Pediatric Cardiology Department National Institute of Cardiology (INC) Rio de Janeiro RJ Brazil; ^4^ Liver Transplant Surgery Department Rede D'Or Rio de Janeiro RJ Brazil; ^5^ Radiology Department, Federal University of Rio de Janeiro (UFRJ) Rio de Janeiro RJ Brazil; ^6^ Institute for Studies in Public Health—IESC Federal University of Rio de Janeiro (UFRJ) Rio de Janeiro Brazil; ^7^ Fluminense Federal University Niterói RJ Brazil; ^8^ Internal Medicine Department Federal University of Rio de Janeiro (UFRJ) Rio de Janeiro RJ Brazil; ^9^ Pediatric Cardiac Transplantation National Institute of Cardiology (INC) Rio de Janeiro RJ Brazil

**Keywords:** combined heart‐liver transplantation, Fontan, Fontan‐associated liver disease, liver fibrosis, mortality

## Abstract

**Background:**

Fontan‐associated liver disease can progress to advanced fibrosis, raising the potential need for combined heart–liver transplantation (CHLT) in selected patients. However, the benefits of CHLT over isolated orthotopic heart transplantation (HT), particularly in terms of mortality, remain uncertain. In this systematic review, we compared mortality outcomes following CHLT versus HT in patients with Fontan circulation, with the aim of supporting clinical decision‐making.

**Methods:**

This systematic review was conducted according to the 2020 PRISMA guidelines and registered in PROSPERO. PubMed, Scopus, and Embase were searched. Studies examining HT or CHLT in patients with Fontan circulation that provided information about total and/or 1‐year mortality were included. Bias risks were assessed using the Newcastle‐Ottawa Scale. We used random‐ and fixed‐effect models, depending on heterogeneity, to estimate pooled effects.

**Results:**

Sixteen studies were included in this analysis. CHLT was associated with a lower mortality rate per patient‐year compared to HT (0.03 vs. 0.09; *p* < 0.01). However, after excluding studies in which transplantations were performed before the year 2000, the difference between groups was no longer statistically significant. One‐year mortality rates were also not significantly different between CHLT and HT (0.09 vs. 0.14; *p* = 0.28), with similar results observed after excluding pre‐2000 studies.

**Conclusion:**

Overall, this systematic review suggests that CHLT may result in mortality rates comparable to those of isolated HT. These findings support the consideration of CHLT in patients with concomitant liver disease and reinforce the importance of comprehensive liver evaluation in transplant candidates.

## Graphical abstract

This meta‐analysis compares mortality rates in Fontan patients undergoing combined heart–liver transplantation (CHLT) versus isolated heart transplantation (HT), showing comparable mortality and reinforcing the importance of comprehensive liver evaluation in transplant candidates.

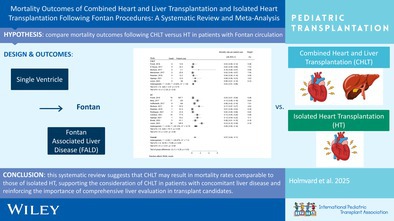

**Abbreviations**
CHD: congenital heart diseases
CHLT: Combined heart‐liver transplantation
FALD: Fontan‐associated liver disease
HT: Orthotopic heart transplantation
NOS: Newcastle‐Ottawa Scale
PLE: Protein‐losing enteropathy
VAST: varices, ascites, splenomegaly and thrombocytopenia

## 1 Introduction

The Fontan procedure is a palliative surgical approach designed for patients with univentricular hearts [1, 2, 3]. Chronically elevated systemic venous pressure and reduced cardiac output can lead to various complications including Fontan associated liver disease (FALD) [4, 5, 6, 7, 8, 9]. FALD results from Fontan physiology that may lead to sinusoidal dilatation and hepatocellular injury, progressing to fibrosis and cirrhosis [10], with an increasing prevalence estimated at 52.24% in 35 years post‐procedure [6].

Orthotopic heart transplantation (HT) is the conventional treatment for end‐stage complications in Fontan [11, 12]. For patients with liver fibrosis or cirrhosis, some centers with extensive experience in multiple organ transplantation offer the possibility of combined heart and liver transplantation (CHLT); however, a clear consensus on which patients would benefit from this combined approach is lacking [4].

The comparative evaluation of these approaches is essential to optimize treatment strategies and improve survival in this population. Thus, this systematic review and meta‐analysis was performed to evaluate and compare the mortality of patients with Fontan circulation following HT and CHLT as the primary outcome.

## 2 Patients and Methods

This systematic review was conducted according to the 2020 PRISMA reporting guidelines and was registered in PROSPERO (CRD42024603026). The completed PRISMA checklist is available in the Supporting Information. In September 2024, we searched PubMed, Scopus, and Embase databases using the following key words: Fontan (or univentricular physiology or single ventricle) and transplants (or transplantation or transplant) and liver (or hepatic). The complete search strategy is described in the Supporting Information. Manual searches of the reference lists of key articles, reviews, and guidelines were conducted to identify additional relevant studies.

The inclusion criteria were: (1) randomized or observational studies and (2) inclusion of patients with Fontan circulation who underwent HT or CHLT. In cases of patient population overlap, the publication with the most comprehensive liver‐related data was included. Exclusion criteria were: (1) mixed population preventing the distinction of outcomes for patients with Fontan circulation; (2) review article, meta‐analysis, case report, poster, or comment; and (3) publication in a language other than English.

Two independent blinded reviewers screened the titles and abstracts of retrieved publications, applying the inclusion and exclusion criteria using Rayyan software [13]. Two reviewers extracted data using predefined criteria, and conflicts were resolved by consensus.

### 2.1 Outcomes

The primary outcomes assessed were total mortality, measured as mortality per patient‐year, and 1‐year mortality. Patient‐year mortality was calculated as a product of the median follow‐up time and the total number of patients with Fontan circulation included in the study. Secondary outcomes assessed were liver abnormalities, based on clinical and laboratory data, non‐invasive imaging, and biopsy findings. For each pooled data analysis, studies were included only if they reported the number of events and the time of follow‐up or 1‐year mortality.

### 2.2 Analysis of Bias

The methodological quality of the included studies was assessed using the Newcastle‐Ottawa Scale (NOS), which characterizes the risk of bias of non‐randomized studies in three main domains: selection (0–4 stars), comparability (0–1 star), and outcome (0–3 stars). Two independent reviewers conducted the assessment, with disagreements resolved by consensus.

Publication bias was assessed with funnel plots and Egger's test.

### 2.3 Statistical Analysis

We conducted a meta‐analysis to evaluate the overall mortality rates per patient‐year for individuals who underwent CHLT and HT using a proportional model. The pooled 1‐year mortality rate was estimated using a fixed‐ or random‐effects model, depending on the degree of heterogeneity (variation in outcomes among studies), which was assessed using the *I* ^2^ statistic and Cochrane *Q* test. Sensitivity analyses were performed to evaluate the robustness of the findings and address sources of heterogeneity. All statistical analyses were conducted using STATA (version 18; StataCorp LLC, College Station, TX, USA).

## 3 Results

**FIGURE 1:**
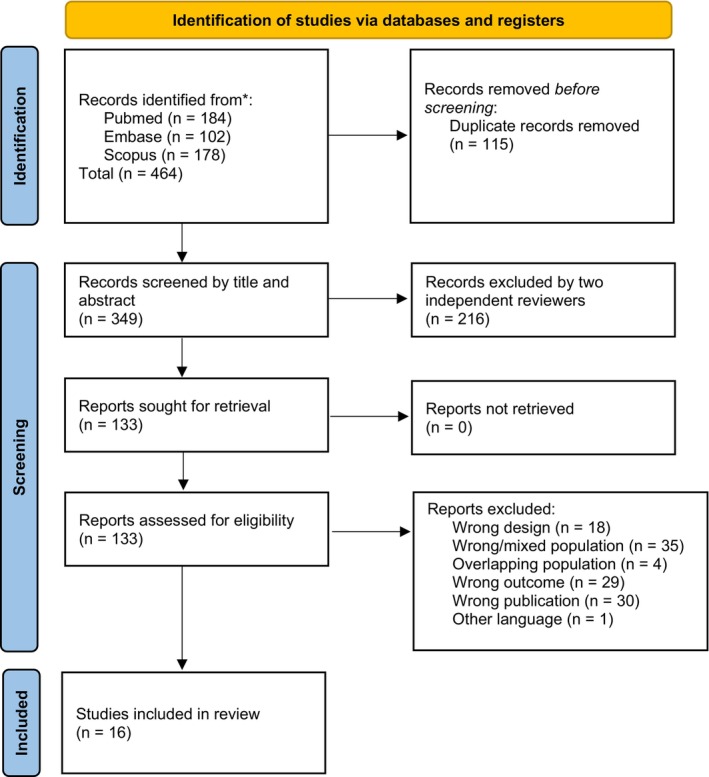
PRISMA flowchart of study selection.

Our search conducted in September 2024 identified 464 studies. After removing 115 duplicate records, 349 studies remained. Following title and abstract screening, 216 articles were excluded, leaving 133 studies for full‐text retrieval, all of which were available for eligibility assessment. Ultimately, 16 studies met the inclusion criteria: 5 focused solely on HT [14, 15, 16, 17, 18], 3 on CHLT patients [19, 20, 21], and 8 on both HT and CHLT (Figure 1) [22, 23, 24, 25, 26, 27, 28, 29]. All included studies were observational or case series.

In total, data from 467 patients with failing Fontan physiology and/or FALD from 16 studies were included in this analysis. Sample sizes ranged from 9–131 patients; 373 (79.8%) patients underwent HT and 94 (20.2%) patients underwent CHLT. Patient mean ages ranged from 13–40 years, reflecting the diversity of the cohort. Balanced sex distributions were reported for most studies. Additional studies' characteristics are summarized in Table 1.

**TABLE 1:** Characteristics of studies included in the meta‐analysis.

Author year	Design	Patients (*n*)	CHLT (*n*)	HT (*n*)	Male (%)	Age (year)	Follow‐up time (year)	LOS (days)	Age fontan (year)	Fontan duration (year)	ICU time (days)
Simpson 2014 [14]	Cohort	20	0	20	—	13 ± 6.4	—	—	—	8.5 (3–15)	—
Pundi 2016 [22]	Cohort	44	1	43	—	23.4 ± 12	13.9 ± 10.1	—	9.9 ± 7.7	13.0 ± 7.7	—
D'Souza 2017 [23]	Cohort	10	7	3	53%	36.8 [27.3–41.7]	4.6 [0.6–5.8]	29 [25–112]	—	22.9 [18.7–28.5]	—
Berg 2017 [24]	Cohort	36	2	34	41%	21.3 [7.2–48.1]	3.5	23.9 [8–76]	8.3 [2.4–30.3]	—	—
Hofferberth 2017 [15]	Cohort	30	0	30	60%	11.3 [2.6–30.5]	5 [0.25–13]	—	—	7.5 [0.5–17.6]	—
Murtuza 2017 [25]	Cohort	26	1	25	58.8%	26.1 ± 8.6	2.1 [0–20.2]	—	8.6 [± 5.7]	17.7 ± 9.1	—
Menachem 2017 [19]	Cohort	8	8	0	35%	40 [23–57]	3.16	—	—	—	—
Reardon 2018 [26]	Cohort	20	5	15	50%	29.5 [19–44]	4.66	51 [26–77] 23^a^ [8–76]^b^ ^s^	5.5 [3–22]	—	—
Vaikunth 2019 [20]	Cohort	9	9	0	33.3%	20.7 [14.2–41.3]	—	29 [13–197]	—	16.6 [8.4–25.9]	19 [5–96]
Rodriguez 2021 [16]	cohort	9	0	9	—	[10–19]	[1.75–3.5]	[7–48]	—	—	[4–17]
Cardoso 2021 [17]	Cohort	31	0	31	58.1%	27.1 (16.7–53.3)	—	60 (72)	6.9 (2–38)	15.4 ± 7.23	11 (25)
Sganga 2021 [27]	Cohort	47	9	38	62%	15 (11–20)	1.4 (0.4–4.3)	28.5 (17–47)	—	—	9.5 (6–20)
Broda 2022 [18]	Cohort	14	0	14	30%	36 (30–48)	4.7 (1.4–8.8)	—	—	31 (23–33)	—
Rezkalla 2022 [28]	Cohort	21	10	11	61%	35.2 [18–66]	—	—	—	—	—
Lewis 2023 [29]	Cohort	131	40	91	52%	30 ± 9.3	1.6 (0.35–4.3)	25 (17–50)	—	23 ± 7.9	11 (6–22)
Wu 2024 [21]	Cohort	11	11	0	63.3%	37 (30–48)	—	26 [16–32]	7.0 (3.0–17.0)	—	7.5 (7–13)

*Note:* Values are described as mean ± SD or median (IQR) or [range].

^a^ CHLT combined heart‐liver transplantation.

^b^ HT heart transplantation.

LOS length of stay (in the hospital), ICU intensive care unit.

### 3.1 Mortality per Patient‐Year

**FIGURE 2:**
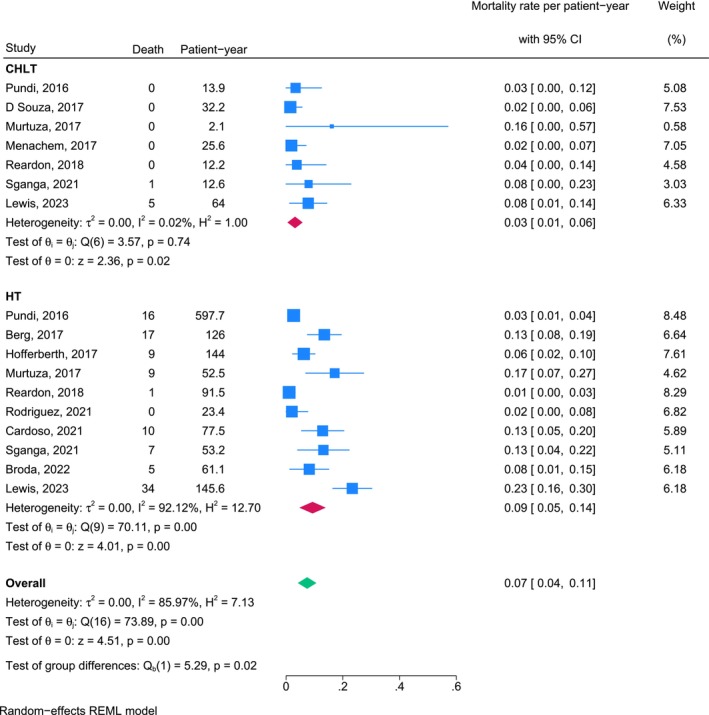
Overall mortality rates per patient‐year for patients who underwent CHLT and HT. CHLT combined heart‐liver transplantation, HT heart transplantation, REML, Restricted Maximum Likelihood.

The mortality rate per patient‐year for CHLT cohorts varied among studies, with a pooled estimate of 0.03 (95% CI, 0.01–0.06) and minimal heterogeneity (*I* ^2^ = 0.02%). The mortality rate per patient‐year for HT cohorts was higher (0.09; 95% CI, 0.05–0.14), with considerable variability of outcomes among studies (*I* ^2^ = 92.12%). The overall pooled mortality rate across all studies was 0.07 (95% CI, 0.04–0.11); this rate was obtained using a random‐effects model due to significant heterogeneity (*I* ^2^ = 85.97%). CHLT was consistently associated with lower mortality rates per patient‐year compared with HT (*p* = 0.02), highlighting a potential survival advantage (Figure 2).

**FIGURE 3:**
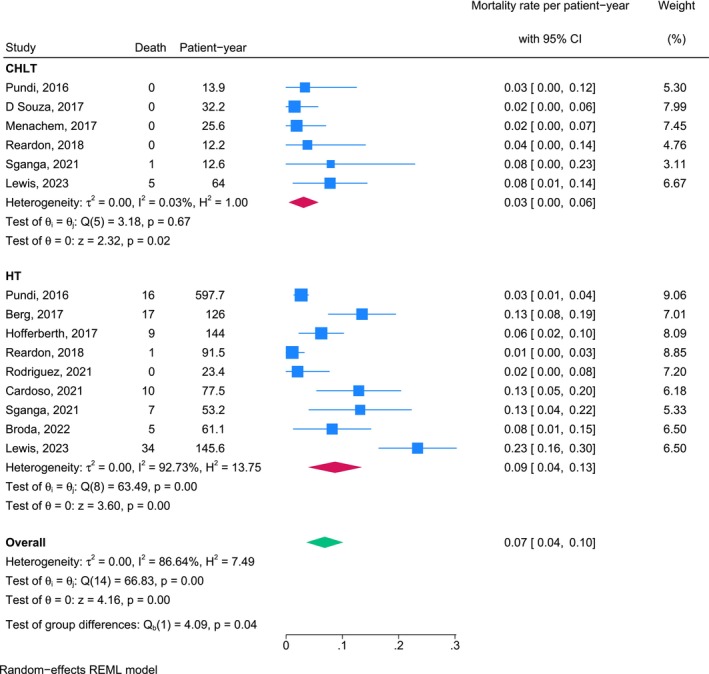
Sensitivity analysis results for overall mortality rates per patient‐year for patients who underwent CHLT and HT. CHLT combined heart‐liver transplantation, HT heart transplantation, REML, Restricted Maximum Likelihood.

Sensitivity analyses confirmed the consistency of mortality rates per patient‐year after the exclusion of data from one study that included a single patient who underwent CHLT and yielded a wide CI [25]. The updated pooled mortality rate for the CHLT cohort remained 0.03 (95% CI, 0.00–0.06), and that for the HT cohort was 0.09 (95% CI, 0.04–0.13). The overall pooled mortality rate per patient‐year across all studies, obtained using a random‐effects model, was 0.07 (95% CI, 0.04–0.10). After this adjustment, the mortality rates per patient‐year associated with CHLT remained consistently lower than those associated with HT (*p* = 0.04; Figure 3).

**FIGURE 4:**
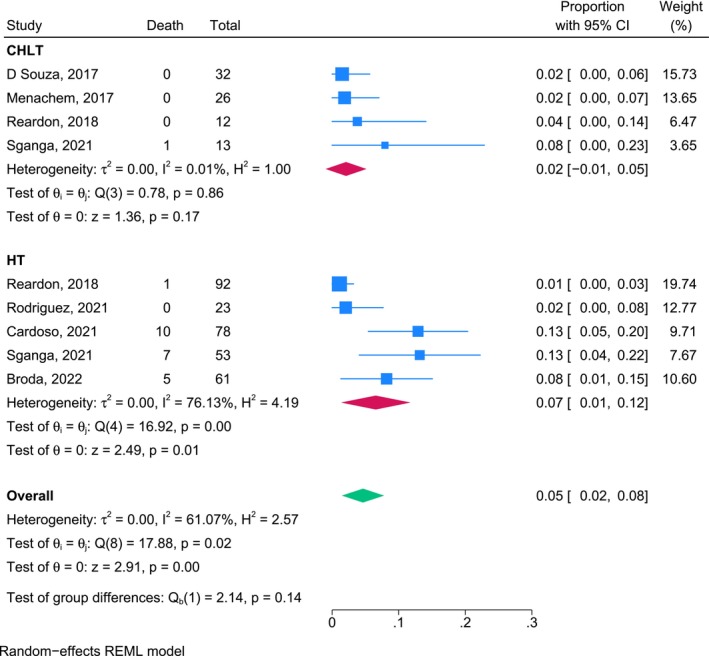
Sensitivity analysis results for overall mortality rates per patient‐year for patients who underwent CHLT and HT from the year 2000 onward. CHLT combined heart‐liver transplantation, HT heart transplantation, REML, Restricted Maximum Likelihood.

We conducted a sensitivity analysis with the exclusion of studies in which transplantations were performed before the year 2000. The updated pooled mortality rate per patient‐year for the CHLT cohort was 0.02 (95% CI, −0.01 to 0.05) and that for the HT cohort was 0.07 (95% CI, 0.01–0.12). The overall pooled mortality rate across all studies, calculated using a random‐effects model, was 0.05 (95% CI, 0.02–0.08). No significant difference in the mortality rate per patient‐year was observed between groups (*p* = 0.14; Figure 4).

### 3.2 One‐Year Mortality

**FIGURE 5:**
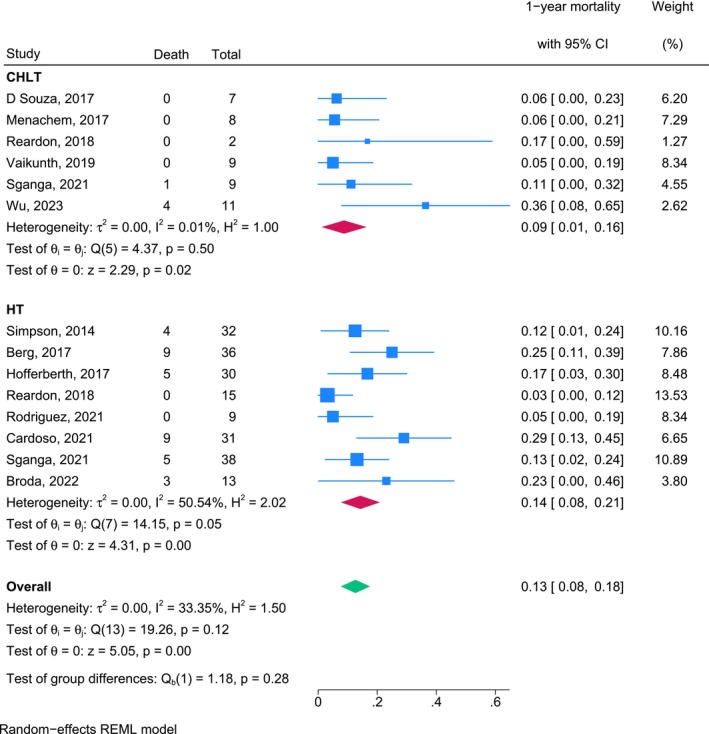
One‐year mortality rates for patients who underwent CHLT and HT. CHLT combined heart‐liver transplantation, HT heart transplantation, REML, Restricted Maximum Likelihood.

The 1‐year mortality rate for patients who underwent CHLT was 0.09 (95% CI, 0.01–0.16), and that for patients who underwent HT was 0.14 (95% CI, 0.08–0.21). The overall pooled 1‐year mortality rate across all studies was 0.13 (95% CI, 0.08–0.18). Overall, 33.5% heterogeneity was observed among studies; heterogeneity was negligible in the CHLT group (*I* ^2^ = 0.00%) and moderate in the HT group (*I* ^2^ = 50.54%). No significant difference in the 1‐year mortality rate was found between the CHLT and HT groups (*p* = 0.28; Figure 5).

**FIGURE 6:**
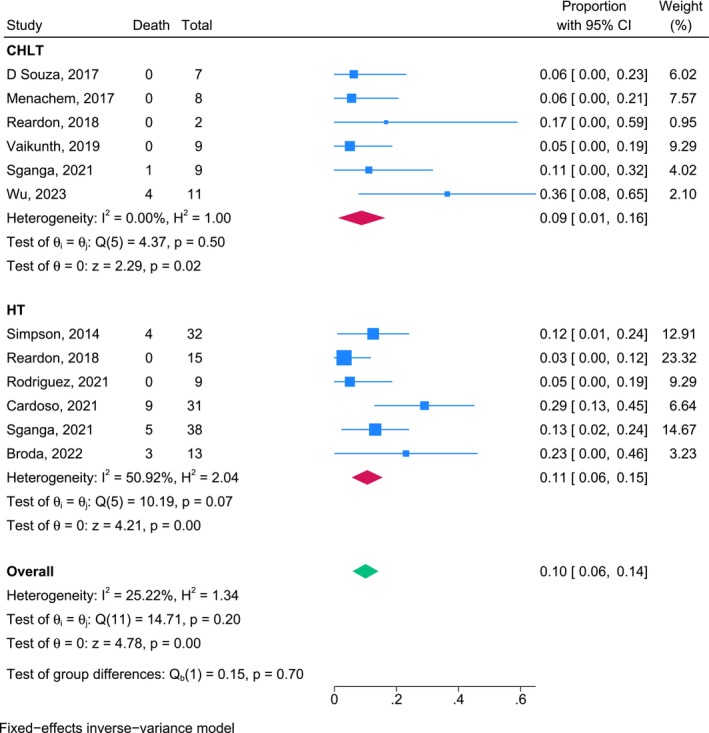
Sensitivity analysis results for 1‐year mortality rates for patients who underwent CHLT and HT from the year 2000 onward. CHLT combined heart‐liver transplantation, HT heart transplantation, REML, Restricted Maximum Likelihood.

A sensitivity analysis of 1‐year overall mortality with the exclusion of studies in which transplantations occurred before the year 2000, performed using a fixed‐effects inverse‐variance model, yielded pooled estimates of 0.09 (95% CI, 0.01–0.16) for the CHLT cohort and 0.11 (95% CI, 0.06–0.15) for the HT cohort. The overall pooled 1‐year mortality estimate for post‐2000 transplantations was 0.10 (95% CI, 0.06–0.14; Figure 6).

Cardiac outcomes including primary graft dysfunction, graft rejection, coronary allograft vascular disease, and causes of death were described in Table S1.

### 3.3 Qualitative Liver Assessments

#### 3.3.1 Clinical and Laboratory Assessment

Ascites prevalence ranged from 5% to 90% [16, 18, 20, 21, 23, 26, 27, 29]. Lewis et al. [29] and Vaikunth et al. [20] reported splenomegaly in 53% and 44% of patients, respectively. Esophageal varices occurred in 14.3%–54.6% [18, 20, 21, 23, 27, 29].

MELD‐XI scores ranged from 6.8 to 14.2 [15, 16, 17, 18, 20, 21, 23, 25, 26, 27, 28]. Sganga et al. [27] reported no MELD‐XI difference between groups, and Hofferberth et al. [15] noted mild liver dysfunction without correlation to architectural distortion. Rodriguez et al. [16] reported successful short‐term HT outcomes in patients with MELD‐XI < 16 and VAST scores ≤ 2. Berg et al. [24] reported a higher mortality with MELD‐XI > 19, and Murtuza et al. [17] reported higher MELD‐XI in non‐survivors.

Reardon et al. [26] and Vaikunth et al. [20] reported that most CHLT patients had VAST scores > 2, linked to a higher number of adverse outcomes; Wu et al. [21] reported a median VAST of 2 for CHLT. Sganga et al. [27] found higher Child–Pugh and VAST scores in CHLT versus HT. Murtuza et al. [25] found no VAST, ultrasound, or albumin differences by survival.

Simpson et al. [14] found a 47% prevalence of PLE with no difference in patients with or without cirrhosis; Pundi et al. [22] reported an association between PLE and post‐transplant mortality; however, Berg et al. [24] and Cardoso et al. [17] found no association with mortality or complication. D'Souza et al. [23] identified PLE as the main CHLT indication, while Sganga et al. [27] reported similar PLE prevalence in CHLT and HT.

#### 3.3.2 Non‐Invasive Imaging Findings

Hofferberth et al. [15] reported a high prevalence (73%) of ultrasound liver abnormalities before HT but found no difference in 1‐year survival among groups. Similarly, Broda et al. [18] reported a high prevalence of ultrasound abnormalities, with evidence of liver cirrhosis in 57% of patients. Cirrhosis was reported in 18%–100% of patients [14, 20, 22, 23, 27, 29]. Rodriguez et al. [16] reported the persistence of nodule enhancement on MRI in two patients after HT.

#### 3.3.3 Biopsy

Pre transplantation liver biopsies consistently confirmed cirrhosis, particularly in patients who underwent CHLT [20, 21, 23, 26, 27, 28]. D'Souza et al. [23] reported that biopsy findings correlated with intraoperatively analysis.

Lewis et al. [29] observed stage‐4 fibrosis or cirrhosis in 18% of patients. Sganga et al. [27] identified stage‐3 fibrosis in 50% of patients in CHLT and 33% in HT groups. Reardon et al. [26] noted cirrhosis in 29.4% of patients, and recommended CHLT for them.

Broda et al. [18] reported abnormal biopsy findings for all six patients, including five with cirrhosis. Vaikunth et al. [20] reported that all patients in their CHLT group who underwent biopsy had bridging fibrosis, with all explant analyses confirming cirrhosis. Similarly, Wu et al. [21] and Hofferberth et al. [15] observed fibrosis in all biopsied patients, with cirrhosis rates of 28.6% and 33.3%, respectively. Rezkalla et al. [28] detected cirrhosis in 71% of biopsied cases.

Rodriguez et al. [16] evaluated paired pre‐ and post‐transplantation biopsy samples and found no significant change in liver fibrosis scores within 12 months, reflecting no regression of the fibrotic process after HT.

### 3.4 Bias Analysis

Funnel plots (Figure S1) demonstrated a slight rightward asymmetry, which may indicate the presence of publication bias. Egger's test for small‐study effects showed a significant probability of bias for mortality per patient‐year (*p* = 0.03) and no bias for 1‐year mortality (*p* = 0.31). Most included studies received high NOS scores in the selection domain, with several studies achieving the maximum of four stars. Scores in the comparability domain were generally lower, with most studies receiving zero or one star. Outcome domain scores were distributed more evenly, with a few studies attaining the maximum of three stars (Table S2).

## 4 Discussion

The present systematic review and meta‐analysis included 16 studies and was performed to compare the proportions of deaths occurring after CHLT and HT alone in patients with Fontan circulation. Despite the additional complexity of the surgical procedure in patients who underwent CHLT, our findings showed similar patient‐year and 1‐year mortality rates in the two groups.

The findings of this meta‐analysis align with those from studies including patients with other congenital heart diseases (CHDs) [30, 31]. In a cohort of 2336 patients with CHD, Bakhtiyar et al. [30] found no difference in 1‐year mortality between CHLT and HT groups, consistent with our results. In an analysis of data from a large US transplant registry, Cannon et al. [31] found no significant difference in mortality following isolated and combined transplantations for CHDs and non‐CHDs, further supporting our findings.

Two previous meta‐analyses yielded survival rates similar to ours for patients with Fontan circulation who underwent isolated HT [32, 33]. Márquez‐González et al. [32] reported increased mortality in patients with chronic kidney disease, but not in those with PLE, heart failure, or arrhythmias; however, among the studies included in the present review, findings regarding the association between PLE and mortality were inconsistent, precluding a definitive conclusion. They also reported a pooled 1‐year survival rate of 79%. Similarly, Tabarsi et al. [33] reported a 1‐year survival rate of 80.3%. These findings align with our results for HT. However, liver function and its impact on outcomes were not assessed in those meta‐analyses. Although Simpson et al. [14] reported no difference in post‐transplantation survival between cirrhotic and non‐cirrhotic patients, other studies have shown that isolated HT is associated with worse outcomes in patients with liver dysfunction [4], and Sganga et al. [27] reported 67% and 89% 1‐year survival rates for patients with and without cirrhosis, respectively, highlighting the critical need to carefully evaluate the indications for CHLT in this patient population.

### 4.1 Clinical and Laboratory Assessment

Clinical and laboratory findings are often used to assess FALD, and scores such as VAST, MELD, and MELD‐XI provide valuable information about the severity of liver dysfunction, but their reliability in guiding HT versus CHLT decision making is limited. The present data show that laboratory findings, including the MELD‐XI score, may underestimate the structural and functional extent of FALD. Consistently, Hofferberth et al. [15] observed no strong correlation between laboratory markers and the degree of liver architectural distortion. The VAST score is used to evaluate portal hypertension and liver disease severity. In the present analysis, and in accord with Elder et al. [34], VAST scores ≥ 2 were more common in patients who underwent CHLT [20, 21, 26, 27, 35] and were associated with a higher number of adverse events [26].

### 4.2 Non‐Invasive Imaging

Various non‐invasive imaging modalities, including ultrasound with or without elastography, CT, and MRI, have been employed before transplantation to assess liver involvement in patients with Fontan circulation [36, 37]. In the studies included in this meta‐analysis, imaging‐detected liver abnormalities were highly prevalent, with rates of 73% reported for ultrasound [15], 57% reported for CT or MRI [18, 20, 29], and 44% reported for other methods [27]. These findings align with the documentation of liver abnormalities ranging from mild changes to advanced fibrosis in most patients with Fontan circulation [38]. Notably, cirrhosis has been identified in some patients as early as 4 years after the Fontan procedure [39].

In some studies included in this analysis, such as those of Hofferberth et al. [15] and D'Souza et al. [23], imaging findings did not correlate with liver function test results. This observation corroborates previous statements that laboratory tests may be of limited utility for the staging of liver fibrosis in FALD [36, 40]. Chronic hepatic congestion in patients with FALD may cause no or mild liver enzyme elevation, and clotting parameters may be normal, even in the advanced stages of the disease [39].

Advanced MRI techniques, such as elastography and tissue characterization with T1 mapping, have been proposed for the assessment of liver fibrosis and congestion [41], and recent studies support their use for the simultaneous assessment of the heart and liver [37]. Despite these advancements, the present results underscore that although liver abnormalities are quite prevalent in this population, clear imaging‐based criteria defining the best approach for patients with FALD (i.e., regarding the need for liver transplantation) have not been established. Indications for liver transplantation in patients with failing Fontan physiology are not uniform. For instance, the 2015 American College of Cardiology conference report [38] states that only patients with strong evidence of cirrhosis require concomitant liver transplantation, but no strategy for the following of these patients or the stratification and prediction of the risk of liver fibrosis progression has been established.

### 4.3 Biopsy

Silva‐Sepulveda et al. [42] evaluated the relationship between magnetic resonance elastography and transjugular liver biopsy findings and found a positive correlation between liver stiffness and the fibrosis stage. Liver biopsy is still considered to be the gold standard for the assessment of fibrosis and cirrhosis in patients with Fontan circulation, but it is an invasive procedure, and this population has a high risk of bleeding. In addition, the distribution of fibrosis in the livers of patients with Fontan circulation can be patchy, leading to the potential for sampling error and false‐negative results when biopsy does not capture the most affected areas. The prevalence of biopsy‐proven cirrhosis varied among the studies included in this analysis, and although it was the main indication for CHLT, correlations between biopsy and surgical findings are not entirely consistent [23]. Even so, D'Souza et al. [23] recommended the performance of liver biopsy within 1 year before CHLT.

Rodriguez et al. [16] reported no regression of liver fibrosis 12 months after isolated HT, showing that the underlying hepatic vascular remodeling and fibrosis may not fully resolve with the restoration of normal cardiac output and venous pressures and suggesting that the liver changes induced by Fontan physiology are irreversible or slow to regress. These findings are corroborated by other studies that presented heterogeneous liver outcomes post isolated HT [18, 43, 44] Due to its invasive nature, biopsy is not feasible for the monitoring of disease progression. Non‐invasive methods such as multiparametric MRI could be alternatives to biopsy [37], and future longitudinal studies should improve the standardization of non‐invasive imaging protocols, lead to the definition of cutoffs for fibrosis quantification, and provide guidance for treatment strategies for this population.

One possible limitation of this study is potential publication bias, as centers with limited CHLT experience and consequently high short‐term mortality rates may be less inclined to publish their results. Furthermore, our search strategy led to the inclusion only of studies involving liver assessment, and we may have excluded studies of isolated HT. Even so, our results do not differ from those of systematic reviews examining HT alone in patients with Fontan circulation [32, 33].

In addition, advancements in pre‐ and postoperative care and surgical techniques could have significantly impacted outcomes across the studies [45]. However, this limitation was mitigated by performing a sensitivity analysis that excluded studies with cases treated prior to the year 2000.

The growing number of patients with Fontan circulation who survive into adulthood and require transplantation underscores the urgent need to better define management strategies for this population. The development of robust, evidence‐based recommendations for CHLT in patients with FALD remains a challenge due to the unpredictable progression of hepatic fibrosis. This systematic review and meta‐analysis demonstrated that CHLT may not be associated with significantly greater mortality compared to HT, highlighting the importance of routinely performing a comprehensive assessment of liver status prior to transplantation.

## Conflicts of Interest

The authors declare no conflicts of interest.

## Supporting information


**Appendix S1:** Supporting information.

## Data Availability

The data that support the findings of this study are available from the corresponding author upon reasonable request.
