# Supplementary material for: Mortality Outcomes of Combined Heart and Liver Transplantation and Isolated Heart Transplantation Following Fontan Procedures: A Systematic Review and Meta‐Analysis
Source: Pediatr Transplant. 2025 Sep 7;29(7):e70174. doi: 10.1111/petr.70174 (PMC12415505; doi:10.1111/petr.70174)
Supplement: Supplementary file 1 — Appendix S1: Supporting information. [file PETR-29-e70174-s001.docx]

**Supplementary material**

**Mortality Outcomes of Combined Heart and Liver Transplantation and Isolated Heart Transplantation Following Fontan Procedures: A Systematic Review and Meta-Analysis**

Oscar Holmvard^1^* MD; Mariana Póvoa-Corrêa^1,2^* MD; Adriana M Innocenzi^1,3^ MD, PhD; Lucio F Pacheco^1,4^ MD, PhD; Daniella B Parente^1,5^ MD, PhD; Ronir R Luiz^1,6^ PhD, Jessica P L Moreira^1,7^ PhD; Renata M Perez^1,8^ MD, PhD; Fernanda P Fernandes^1,9^ MD; Renata Moll-Bernardes^1**^ MD, PhD

¹ D’Or Institute for Research and Education (IDOR), Rio de Janeiro, RJ, Brazil
² Maternal and Child Health Department, Federal University of Rio de Janeiro (UFRJ), Macaé, RJ, Brazil
³ Pediatric Cardiology Department, National Institute of Cardiology (INC), Rio de Janeiro, RJ, Brazil

^4^ Liver Transplant Surgery Department, Rede D’Or, Rio de Janeiro, RJ, Brazil

^5^ Radiology Department, Federal University of Rio de Janeiro (UFRJ), Rio de Janeiro, RJ, Brazil

^6^ Institute for Studies in Public Health—IESC, UFRJ, Rio de Janeiro, Brazil

^7^ Fluminense Federal University, UFF, Niterói, RJ, Brazil

^8^ Internal Medicine Department, Federal University of Rio de Janeiro (UFRJ), Rio de Janeiro, RJ, Brazil

^9^ Pediatric Cardiac Transplantation, National Institute of Cardiology (INC), Rio de Janeiro, RJ, Brazil

*These authors contributed equally to this work.

**Corresponding author: Renata Moll-Bernardes. D’Or Institute for Research and Education (IDOR), Diniz Cordeiro, 30, Botafogo, Rio de Janeiro, RJ 22281-100, Brazil.

E-mail address: [renata.moll@idor.org](mailto:renata.moll@idor.org). https://orcid.org/0000-0001-8587-7319

**Search strategy**

***Pubmed***

((((fontan) OR (single ventricle)) OR (univentricular physiology)) AND (((transplant[Title/Abstract]) OR (transplants[Title/Abstract])) OR (transplantation[Title/Abstract]))) AND ((liver) OR (hepatic))

***Scopus***

( ( ALL ( fontan ) OR ALL ( "single ventricle" ) OR ALL ( "univentricular physiology" ) ) ) AND ( ( ABS ( transplant ) OR ABS ( transplants ) OR ABS ( transplantation ) ) ) AND ( ( ALL ( liver ) AND ALL ( hepatic ) ) ) AND ( LIMIT-TO ( SUBJAREA , "medi" ) ) AND ( LIMIT-TO ( DOCTYPE , "ar" ) ) AND ( LIMIT-TO ( LANGUAGE , "english" ) OR LIMIT-TO ( LANGUAGE , "french" ) )

***Embase***

Fontan OR (univentricular AND physiology) OR (single AND ventricle) AND (transplant OR transplants OR transplantation) AND (liver OR hepatic) AND ('Article'/it OR 'Article in Press'/it OR 'Conference Abstract'/it OR 'Letter'/it) AND ('congenital heart disease'/dm OR 'dextrocardia'/dm OR 'heart disease'/dm OR 'heart single ventricle'/dm OR 'heterotaxy syndrome'/dm OR 'hypoplastic left heart syndrome'/dm OR 'protein losing gastroenteropathy'/dm OR 'pulmonary valve atresia'/dm OR 'tricuspid valve atresia'/dm)

**Table S1** Major complications including primary graft dysfunction, graft rejection, coronary allograft vascular disease and causes of death

| **Study** (patient *n*) | **Major complications** |
| --- | --- |
| Simpson et al., 2014^14^  HT (n = 20) | Death  Patients with cirrhosis: massive intraoperative bleeding (3 months; n=1), heart rejection (15 months; n=1)  Without cirrhosis: heart rejection (< 1 year; n = 2); CMV infection (< 1 year; n = 1) |
| D’Souza et al., 2017^23^  CHLT (n = 7), HT (n =3) | Liver rejection resolved with steroids (< 1 year; n = 1), no cardiac rejection > 1R‡ |
| Berg et al., 2017^24^  CHLT (n = 2), HT (n =34) | Perioperative death  Primary graft dysfunction (n = 2), hemorrhage (n = 3), sepsis (n = 2), superior vena cava syndrome (n = 1)  Late death  CAVD (n= 3), heart rejection (n =2), PTLD (n = 1), unknown causes (n = 3). |
| Hofferberth et al., 2017^15^  HT (n =30) | Perioperative death  Multiorgan failure (n = 1), heart rejection (n = 1), stroke (n = 1)  Late death  Heart rejection (n = 1), postoperative tracheoplasty (n = 1), heart failure (n = 1), sudden death (n = 2), unknown cause (n = 1) |
| Murtuza et al., 2017^25^ CHLT (n = 1), HT (n =25) | Perioperative death  Hemorrhage (n = 1), primary graft dysfunction (n = 1), multiorgan failure (n = 1), nonspecified cause (n = 5) |
| Reardon et al., 2018^26^  CHLT (n = 5), HT (n =15) | Late death  CAVD (n = 1, HT)  Hepatic rejection (n = 1)  No heart rejection > 1R  LOS 23 days (HT) and 51 days (CHLT) |
| Vaikunth et al., 2019^20^  CHLT (n = 9), HT (n =0) | Late death  PTLD (n = 1) |
| Cardoso et al., 2021^17^  HT (n =31) | Perioperative death (n = 9)  Late death (n = 1)  Primary graft dysfunction (n = 8) |
| Sganga et al., 2021^27^  CHLT (n = 9), HT (n =38) | Perioperative death  CHLT: Stroke (n = 1)  HT: Multiorgan failure (n = 2), FV during dialysis (n = 1), massive cerebral hemorrhage (n = 1)  Late death (n =3)  Heart rejection (HT; n = 12 and CHTL; n = 0)  Acute hepatic rejection (n = 1) |
| Broda et al., 2022^18^  HT (n = 14) | Perioperative death (n = 3)  Late death  Sudden death (n =1)  Infection (n = 1) |
| Lewis et al., 2023^29,48^† CHLT (n = 40), HT (n = 91) | Perioperative death (n = 15)  Late death (n = 24)  Causes of death: multiorgan failure (n = 11), hemorrhage (n =7), primary graft dysfunction (n =5), heart rejection (n = 4), stroke (n = 2), PTLD (n = 1), heart failure (n = 1), CAVD (n = 1), unknown cause (n = 5) |
| Wu et al., 2024^21^  CHLT (n = 11) | Perioperative death (n = 4) |

†This data was obtained in a complementary paper from the same group (REF)

‡ Revised version of the ISHLT grading system

HT heart transplantation, CMV cytomegalovirus, R CAVD, coronary allograft vascular disease, CHLT combined heart liver transplantation, LOS length of stay, PTLD post-transplant lymphoproliferative disorder

**
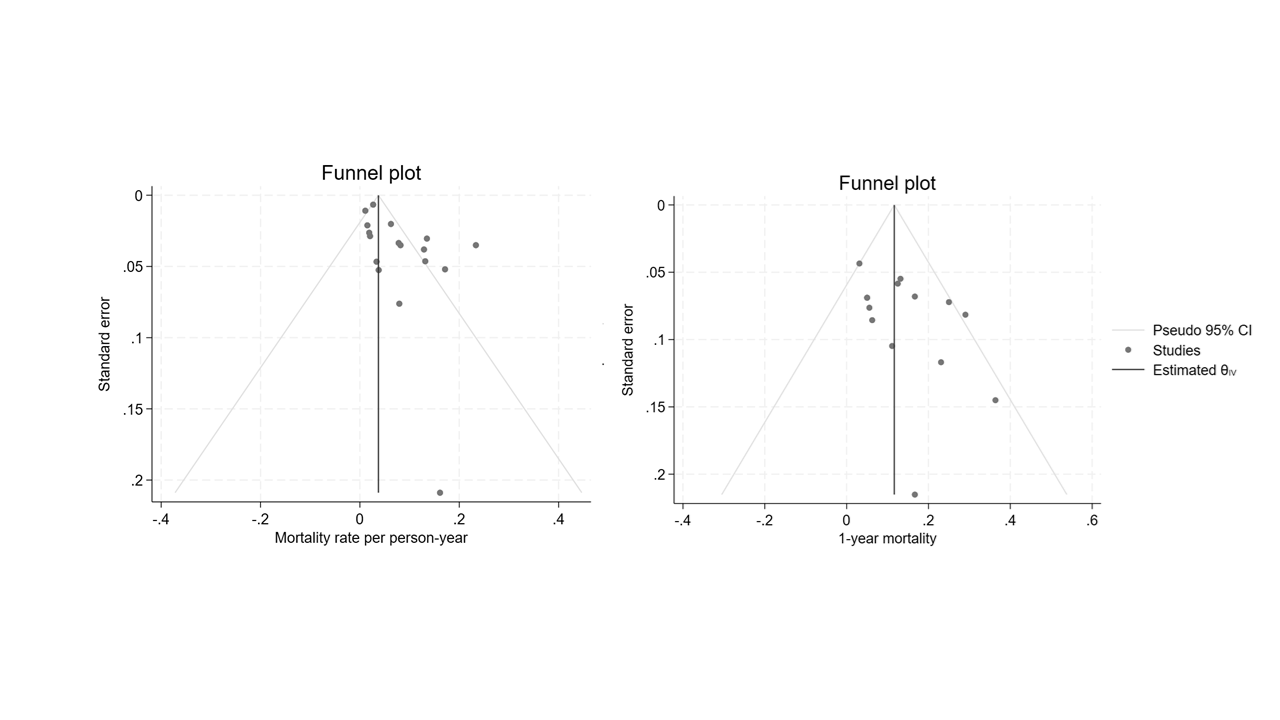
**

**Figure S1** Funnel plots indicating the potential for small-study effects or bias.

**Table S2** Analysis of bias according to Ottawa-Newcastle Scale (NOS) for non-randomized studies.

| **Author, year** | **Selection** | **Comparability** | **Outcome** |
| --- | --- | --- | --- |
| Lewis, 2023^29^ | ******** | ***** | ****** |
| Reardon, 2018^26^ | ****** | - | ****** |
| D’Souza, 2017^23^ | ******* | - | ****** |
| Rodriguez, 2021^16^ | ******* | - | ****** |
| Cardoso, 2021^17^ | ******* | - | ****** |
| Sganga, 2021^27^ | ******** | ***** | ****** |
| Broda, 2022^18^ | ****** | - | ******* |
| Pundi, 2016^22^ | ******** | - | ******* |
| Vaikunth, 2019^20^ | ******* | - | ******* |
| Berg, 2017^24^ | ******* | - | ******* |
| Simpson, 2014^14^ | ******** | - | ******* |
| Wu, 2024^21^ | ******** | - | ******* |
| Rezkalla, 2022^28^ | ******* | - | ******* |
| Hofferberth, 2017^15^ | ******** | - | ******* |
| Murtuza, 2017^25^ | ******** | - | ****** |
| Menachen, 2017^19^ | ****** | - | ******* |

Risk of bias in non-randomized studies, focusing on three main domains: selection (0-4 stars), comparability (0-1 star), and outcome (0-3 stars).
